# Translating CMR Innovations to Impact Cardiovascular Care with a Dedicated Team

**DOI:** 10.1186/1532-429X-18-S1-T3

**Published:** 2016-01-27

**Authors:** Elliot M Hembree, Amy Mumford, Subha V Raman, Kelly J Scheiderer, Anne Garcia, Katherine Bowman, Julie Comyns

**Affiliations:** Ohio State University, Columbus, OH USA

## Background

Typical imaging centers performing cardiovascular magnetic resonance (CMR) examinations involve staff rotating across a variety of procedures, which can limit consistency in proficiency and slow the incorporation of advanced cardiovascular protocols. We describe the design and implementation of a dedicated team to maximize efficiency, competency, and quality in a cardiovascular-dedicated MR center.

## Methods

Administrative tasks needed to create a dedicated team were reviewed. Comparison data on outpatient exams performed 12 months prior and one month after implementation of a dedicated team of nurses along with technologists and physician staff was obtained. Time from arrival at registration to initial image acquisition was recorded for each exam, and staff satisfaction was measured on a 5-point Likert scale (1=highly dissatisfied, 5=highly satisfied). Wilcoxon rank-sum test was used to compare ordinal data.

## Results

**Figure 1:**
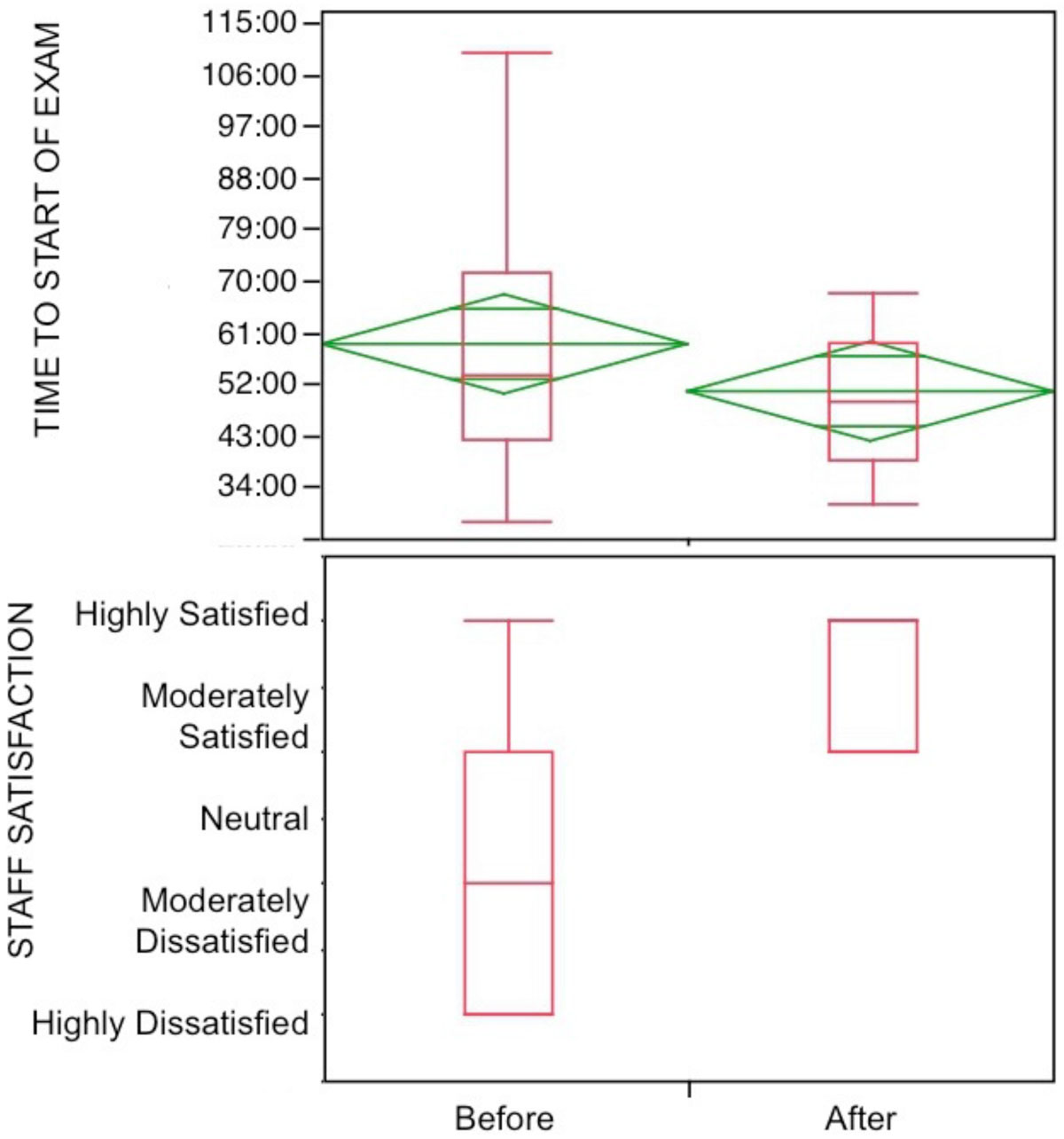
**Top - Time from arrival at registration to start of CMR exam was reduced after implementation of a dedicated team (58 ± 21 vs. 50 ± 15 min, p = 0.09)**. Bottom - Staff satisfaction improved significantly after implementation of the dedicated team model (2 [1-3] vs. 4 [3-4], p < 0.01).

Buy-in was secured over several years of meeting with key stakeholders including hospital physicians, nursing leadership, financial administrators, and existing staff that included 4 technologists and a pool of 12 nurses who rotated daily through various noninvasive labs including CMR. The financial model to support creating an administrative cost center to which dedicated staff could be assigned with the addition of one full-time position was derived from average volume of procedures, reimbursement, payor mix, personnel costs, and supplies/maintenance costs. Volume of procedures was comparable before and immediately after implementation of a dedicated team (8.2 ± 1.6 vs. 7.6 ± 0.9 scans/day, p=NS), while improvements were realized in both throughput (58 ± 21 vs. 50 ± 15 min from registration to start of scan, p = 0.09) as well as staff satisfaction (2 [1-3] vs. 4 [3-4], p < 0.01; Figure 1). Additionally, participants reported subjective observations such as greater comfort level with protocols translating improving knowledge of the required end-result, consistency in results, improved interpersonal relationships with return patients and amongst staff, greater familiarity with cardiovascular emergencies, and greater knowledge of MR safety and relevant pharmacological issues. Facilitated interactions with referring clinicians included review of appropriateness criteria, patient preparation, and care coordination. Ongoing specialized education is planned for continuous quality improvement.

## Conclusions

Creation of a CMR-dedicated staffing team can be done with appropriate organizational buy-in and limited additional resources, improving staff satisfaction and efficiency. Further studies are warranted to assess the impact on study quality and program growth.

